# Study on the potential mechanism of the active components in YiYiFuZi powder in homotherapy for hetropathy of coronary heart disease and rheumatoid arthritis

**DOI:** 10.3389/fchem.2022.926950

**Published:** 2022-08-09

**Authors:** Yuming Wang, Xiaokai Li, Kun Gu, Jing Gou, Xue Li, Yaqian Dong, Rui Li, Jinxia Wei, Zhiying Dou, Yubo Li

**Affiliations:** ^1^ Tianjin University of Traditional Chinese Medicine, Tianjin, China; ^2^ Ningbo Institute of Life and Health Industry, University of Chinese Academy of Science, Ningbo, Zhejiang, China; ^3^ First Teaching Hospital of Tianjin University of Traditional Chinese Medicine, Tianjin, China

**Keywords:** YiYiFuZi powder, coronary heart disease, rheumatoid arthritis, metabolomics, acid sphingomyelinase, atomic force microscope

## Abstract

In recent years, the incidence of coronary heart disease and rheumatoid arthritis has been increasing, which has become a common public health problem worldwide. YiYiFuZi (YYFZ ) powder is a classical traditional Chinese prescription, which is commonly used to treat metabolic diseases such as rheumatoid arthritis, with an ideal curative effect, but the therapeutic mechanism is still unclear. In this study, from the perspective of clinical metabolomics, combined with network pharmacology, we sought the comorbidity mechanism and key targets of coronary heart disease and rheumatoid arthritis and the mechanism by which YYFZ powder exerts therapeutic effects, combined with molecular docking and atomic force microscopy to determine the effective components, and found that the higenamine and steroid components in YYFZ powder can bind acid sphingomyelinase enzymes to affect the sphingolipid pathway to produce therapeutic effects, which can bind to sugars existing as a glycoside.

## 1 Introduction

Coronary heart disease (CHD), a common cardiovascular disease, has become a major health problem in the general population for nearly half a century (Tu et al., 2018). Rheumatoid arthritis (RA) is a common multisystem autoimmune disease involving the peripheral joints of patients, characterized by chronic inflammatory response and progressive bone damage (He et al., 2021). In addition, RA is closely associated with accelerated atherosclerosis and increased incidence of cardiovascular events and death (Dregan et al., 2014; Dregan et al., 2018). Recent pharmacological studies have shown that inflammation and immune regulation are important factors leading to the occurrence and development of both diseases (Curtis et al., 2017). There have been studies on cardiovascular disease risk in RA patients through metabolomics (Niu et al., 2014; De Vecchis et al., 2016) that used transcriptomes to study the molecular mechanisms of inflammation shared by RA and coronary artery disease. However, there are slightly insufficient studies focusing on the metabolic imbalance shared by the two diseases, and few studies have been conducted to find new uses of existing therapeutics for the common metabolic mechanism of the two diseases from the perspective of metabolism.

As an essential part of complementary and alternative medicine, traditional Chinese medicine (TCM) has long been used to treat various metabolic diseases including CHD. From the perspective of TCM, CHD and RA belong to the category of paralysis in TCM. Cold-damp paralysis is an important pathogenic mechanism in the early stage of RA and CHD, which is also an important pathological product in the development of the disease (Wu et al., 2018). YiYiFuZi (YYFZ) powder comes from “Jin Gui Yao Lue,” which is composed of coix seeds and Fuzi. In this formula, coix seeds remove dampness and promote paralysis and guide the downward movement of turbid yin, whereas Fuzi warms yang and disperses cold to remove the paralysis of cold and dampness. The combination of the two herbs, with the combination of warm and cold, can help promote yang and paralysis. coix seed and Fuzi have anti-inflammatory and hypolipidemic effects and have been used to treat CHD and RA, and the application of the two drugs in combination is important for the treatment of patients with CHD and RA (Zhang et al., 2016; Chen, 2019; Wang et al., 2019; Xie, 2019). Studies have shown that the volatile oils and proteins of coix seeds can reduce TNF-α and IL-1 levels in RA patients, thereby reducing RA symptoms and relieving pain (Zhang et al., 2019). Fuzi is commonly used in the treatment of cardiovascular diseases, in which the bis-ester alkaloids are the main potent and toxic substances, producing cardiotonic effects by agonizing myocardial potassium channels. Fuzi polysaccharides can play a role in protecting cardiomyocytes by promoting the synthesis of metallothionein, scavenging reactive oxygen species, counteracting oxidative stress damage, and inhibiting the occurrence of cardiomyocyte apoptosis, as well as suppressing inflammatory symptoms of RA (Tai et al., 2022).

This study aims to investigate the direct and indirect mechanisms of TCM YYFZ powder on the cotreatment of CHD and RA through clinical metabolomics combined with network pharmacology. First, the mechanism of metabolic disorders of the two diseases is explored through clinical metabolomics. Second, through network pharmacology, the common targets and signaling pathways of CHD and RA are collected, analyzed, and integrated. In addition, the common mechanisms of CHD and RA are explored, and the indirect mechanism of how YYFZ powder regulates the disease process of CHD and RA. At last, the main components of YYFZ powder were docked with the joint targets through molecular docking technology to discover the components of YYFZ powder that act directly on metabolic enzymes to exert disease therapeutic effects, and the results were then validated by atomic force. The results were then verified via atomic force microscopic imaging to establish the binding ability of the screened active ingredients to the protein.

## 2 Materials and methods

### 2.1 Instruments and reagents

Formic acid was obtained from ROE Co., Ltd. (United States). Acetonitrile was supplied by Oceanpak Co., Ltd. (Sweden). Pure water was purchased from Watsons (China). Bruker Dimension ICO atomic force microscope (AFM) was obtained from Bruker (Germany). DK-98-II electric-heated thermostatic water bath was provided by Tianjin Taist Instrument Co., Ltd. (China). BT125D 1/100000 electronic analytical balance was purchased from Satorius (Germany). Recombinant human acid sphingomyelinase (ASM) protein was obtained from Shanghai Yubo Biotechnology Co., Ltd. (China). β-sitosterol, higenamine, and stigmasterol reference substances were obtained from Sichuan Vicki Biotechnology Co., Ltd. (China). Dimethyl sulfoxide was supplied by Beijing Solarbio Technology Co., Ltd. (China).

### 2.2 Clinical sample collection

This study included 150 subjects, aged 35–65 years, including 50 cases each in the RA group, CHD group, and normal saline (NS) group. All subjects were collected in the First Teaching Hospital of Tianjin University of Traditional Chinese Medicine from April 2018 to July 2019. After being approved by the Ethics Committee of the First Teaching Hospital of Tianjin University of Traditional Chinese Medicine, data statistics have been performed on the age and clinical performance of each subject, and all subjects signed a patient informed consent.

#### 2.2.1 Inclusion exclusion criteria

Healthy people who met the following conditions were included in the NS group: 1) The result of the physical examination was healthy. 2) There was no metabolic disease or inflammatory disease. 3) The four indicators of rheumatism (anti-hemolytic streptococcal (ASO), C-reactive protein (CRP), rheumatoid factor, and erythrocyte sedimentation rate) were normal.

The inclusion criteria of the RA group are as follows: had more than four joint pains, morning stiffness not less than 30 min, increased platelet count, and increased four indicators of rheumatism; judged that the condition is consistent with the active stage of RA; and enrolled in the RA group as a candidate.

The exclusion criteria of the RA group are as follows: 1) patients with severe joint deformities; 2) patients with other rheumatic diseases such as systemic lupus erythematosus, Sjogren’s syndrome, and knee osteoarthritis; 3) patients with metabolic diseases of the heart, brain, liver, kidney, and other systems; and 4) patients who have taken drugs for the treatment of RA before the study.

The inclusion criteria of the CHD group are as follows: The CRP index of patients in serum biochemical indicators was significantly increased and accompanied by angina pectoris, dizziness, chills, and even syncope, and other clinical symptoms were included in the CHD group as candidates.

The exclusion criteria of the CHD group are as follows: 1) patients with persistent atrial fibrillation, dilated cardiomyopathy, and cardiac insufficiency; 2) patients with severe electrolyte disorders, malignancies, and severe valvular disease; 3) patients with autoimmune diseases; 4) patients with other organic pathologies or other metabolic diseases; and 5) patients who took CHD-improving drugs before the study.

#### 2.2.2 Collection and processing of clinical samples

The serum samples of the subjects were collected and centrifuged at 3,000 * g for 15 min at 4°C. In addition, the supernatant was transferred and centrifuged again at 3,500 * g for 4 min at 4°C, and then the supernatant was stored in a −80°C refrigerator for further serum metabolomics research.

### 2.3 Metabolomics analysis

#### 2.3.1 Sample preparation

After thawing each group of samples frozen in a −80°C refrigerator, the samples were centrifuged at 4°C. A total of 100 μL of supernatant was mixed with acetonitrile in a volume ratio of 1:3, and the mixture was sonicated in an ice-water bath for 10 min and vortexed for 1 min and then centrifuged at 13,200 * g at 4°C. A total of 200 μL of supernatant was transferred into the sample vial for metabolomics analysis.

#### 2.3.2 Instrument conditions

In this experiment, UPLC/Q-TOF-MS (Waters Co., United States) was used to characterize metabolic substances. UPLC separation was performed on the ACQUITY UPLC BEH C18 column (2.1 mm × 100 mm, 1.7 μm, Waters Co., United States), with a column temperature of 45°C. The mobile phase consisted of water (A) and acetonitrile (B) (both containing 0.1% formic acid). The gradient elution program was as follows: 0–0.5 min, 1% B; 0.5–2 min, 1%–50% B; 2–9 min, 50%–99% B; 9–10 min, 99% B; 10–10.5 min, 99%–1% B; and 10.5–12 min, 1% B. The flow rate was 0.3 ml/min. The injection volume was 5 μL.

After separation, an electrospray ion source was used to detect and analyze mass spectrometry in positive and negative ionization modes. The ion source parameters were set as follows: a capillary voltage of 3.0 kV, drying gas temperature of 325°C, atomizing gas pressure of 310 kPa, drying gas flow of 0.26 ml/min, desolvation gas flow of 600 L/h, source temperature of 120°C, desolvation temperature of 350°C, and cone gas flow of 50 L/h.

#### 2.3.3 Data processing

Raw data were exported by MarkerLynx 4.1 (Waters, United States) software, after which the data were imported into SIMCA 14.1 statistical software (Umetrics Corporation, Sweden) for multivariate statistical analysis. Afterward, SPSS 26.0 was applied for statistical tests and the appropriate test was selected to determine whether metabolites changed significantly (*p* < 0.05) in the statistical analysis. The metabolites screened by the above analysis are input into the MetaboAnalyst platform (https://www.metaboanalyst.ca/) for cluster analysis and Mate-Pa analysis.

### 2.4 Network pharmacology analysis

#### 2.4.1 Construction of shared target libraries for coronary heart disease and rheumatoid arthritis diseases

In this study, “Coronary heart disease” and “Rheumatoid arthritis” were used as search terms to search for CHD- and RA-related targets in the CTD (http://ctdbase.org/), TTD (http://bidd.nus.edu.sg/group/cjttd/), durgbank (https://www.drugbank.ca/), and OMIM (http://www.omim.org/) database for retrieval.

The disease targets of the above CHD and RA target libraries were imported into the Venny2.1 (http://bioinfogp.cnb.csic.es/tools/venny/) website to obtain the common targets of the two diseases and draw a Venn diagram.

#### 2.4.2 Visualization and analysis of the relationship network between coronary heart disease and rheumatoid arthritis disease

Import disease common target into STRING (https://string-db.org/DAVID), and obtain the PPI network graph of the interaction between RA-CHD shared targets. Import the PPI map into Cytoscape 3.6.0 to visualize the PPI data between the potential treatment targets shared by the disease on the network.

Import the enriched channel data to the OmicShare cloud platform, and use the advanced bubble chart function to visualize the enriched channel.

#### 2.4.3 Screening of active ingredients and establishment of active ingredient target library for YiYiFuZi powder

The TCMSP search of “Coix seed” and “Fuzi” was used to screen the main active ingredients in coix seed and Fuzi for the active ingredients. The screening of the main active ingredients in the two herbs of YYFZ powder was performed (Yu et al., 2020). Target information of the component actions was obtained from the TCMSP and Swiss Target Prediction (http://www.swisstargetprediction.ch/) websites. The information on the targets of the ingredients from the two websites was aggregated, and the common targets were removed to obtain the active ingredient target library.

#### 2.4.4 Visualization and analysis of active ingredient–target–disease network

PPI maps were created *via* protein interaction analysis through the String website, and PPI maps were imported into Cytoscape 3.6.0 for in-network visualization of PPI data between potential therapeutic targets shared by diseases, and active ingredient–target–disease networks were mapped.

The KEGG pathway enrichment analysis was conducted on the nodes in the network using David’s website to obtain the functional pathways involved in the component action targets of YYFZ powder to modulate diseases, and the enriched pathways were visualized using the advanced bubble map function in OmicShare platform. The results will be compared with the functional pathways involved in the common targets of the two diseases, and a basis for the elaboration of the heterogeneous treatment mechanism of CHD and RA by YYFZ powder will be provided.

### 2.5 Molecular docking

#### 2.5.1 YiYiFuZi Powder components (ligands) and acid sphingomyelinase receptor protein preparation

The 3D structural formulas of the active compounds screened above were saved in .sdf format. The prepared major compounds were bonded into the CHARMM force field with partial charges calculated via the Momany–Rone option (Momany and Rone, 1992). The obtained structures were minimized using the Smart Minimizer algorithm to minimize the conjugation gradient.

#### 2.5.2 Preparation of acid sphingomyelinase receptor protein (5FI9)

In the PDB protein database, the eutectic structure of ASM and inhibitor (ABPA) (PDB ID: 5fi9) was selected as the receptor protein. Import the receptor protein into discovery studio 2017 R2 client software, and prepare the receptor protein in macromolecules. In automatic preparation, click prepare protein. The force field used for calculation was the CHARMM force field.

#### 2.5.3 Verification of molecular docking method

First, the active site was defined for the location of the self-ligand of the 5FI9 protein complex. Second, the self-ligand of the 5FI9 protein complex was extracted for energy optimization, and then, the optimized ligand was docked with the active site of the 5FI9 protein, and the RMSD values of the docked conformation and the conformation in the crystal were calculated if the docked ligand could overlap well with the original crystal ligand structure and the RMSD value was <2. If the docked ligand is in good agreement with the original crystal ligand structure and the RMSD value is <2, then the docking procedure and the selected parameters are suitable for the screening of this study.

#### 2.5.4 Docking operation

The Docking Ligands (CDOCKER) semiflexible docking mode in the receptor–ligand interaction section was selected for molecular docking. The binding activity of the compound to the target is evaluated based on the score and the reasonableness of the bond between the ligand and the amino acid residues of the receptor protein.

### 2.6 Atomic force microscopy

#### 2.6.1 Preparation of solution

A buffer solution with pH 8.0 was prepared with 20 mM Tris and 150 mM NaCl, and ASM protein was prepared to 25 μg/ml of ASM protein solution by adding buffer. Higenamine, β-sitosterol, and stigmasterol were dissolved in DMSO, and the solution was configured to a final concentration of 25 μg/ml by adding buffer (DMSO ≤ 0.1%).

#### 2.6.2 Atomic force microscopy

The 25 μg/ml ASM protein solution and the pure water solvent were mixed 1:1 and added to the newly peeled silicon wafers for crystallization for 15 min, rinsed with ultrapure water, dried, and put into the AFM sample table for imaging. Mix 25 μg/ml of ASM protein solution and 25 μg/ml of standard solution 1:1, incubate for 30 min at room temperature, then add the solution to the silicon wafer for crystallization, rinse with ultrapure water to dry, and then put it into the AFM sample stage for imaging. The AFM was imaged in tap mode under gas phase room temperature conditions.

## 3 Result

### 3.1 Metabolomics analysis results

#### 3.1.1 Screening and identification of coronary heart disease and rheumatoid arthritis common biomarkers

**FIGURE 1:**
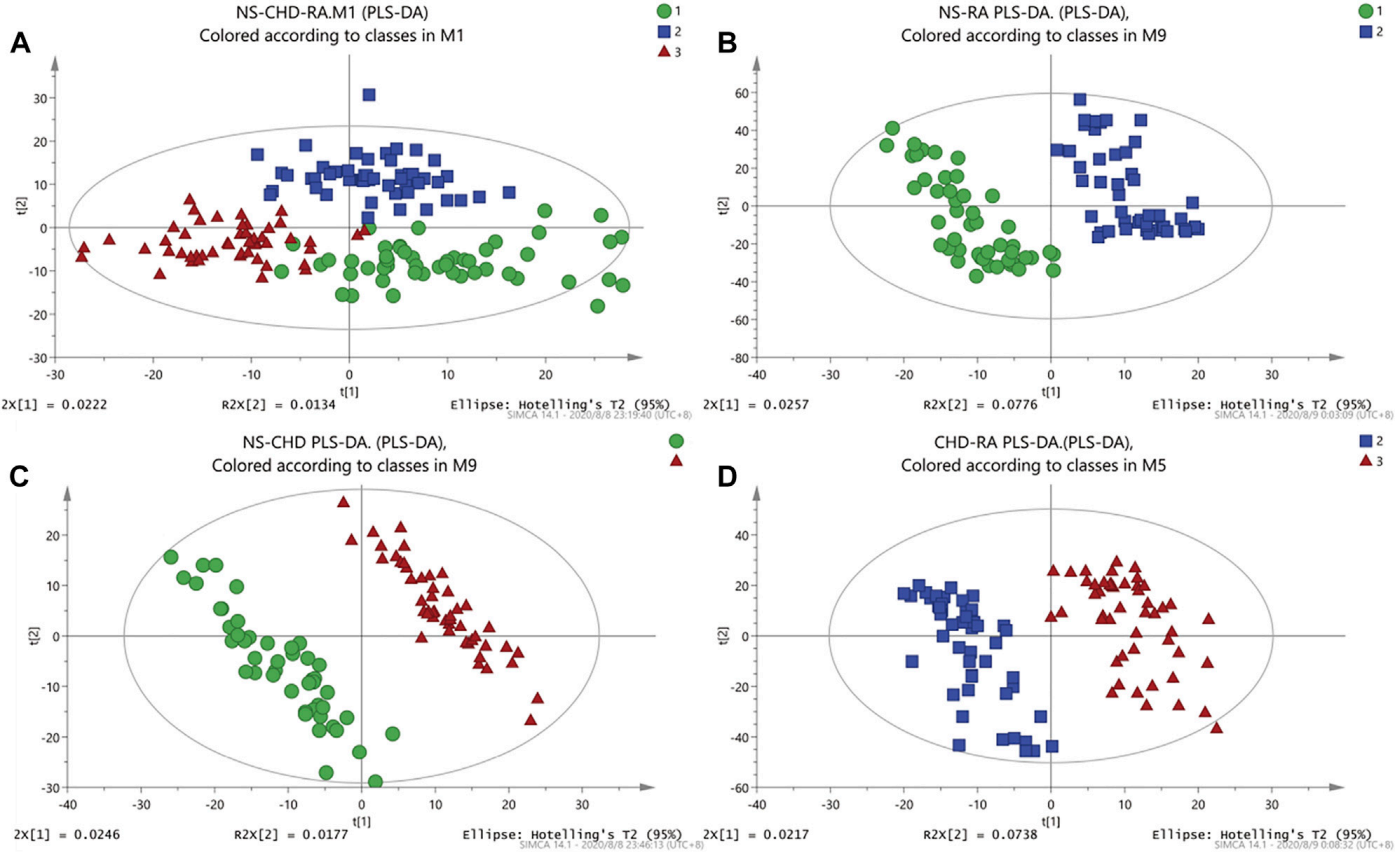
PCA, PLS-DA diagram of rheumatoid arthritis (RA), coronary heart disease (CHD), and NS group. **(A)** NS-CHD-RA three groups of PLS-DA diagrams (R2X = 0.0324, R2X = 0.762, Q2 = 0.249); **(B)** NS-RA group of PLS-DA diagrams (R2X = 0.13, R2Y = 0.915, Q2 = 0.579); **(C)** PLS-DA diagram of NS-CHD group (R2X = 0.0482, R2Y = 0.956, Q2 = 0.415); **(D)** PLS-DA diagram of CHD-RA group (R2X = 0.0955, R2Y = 0.903, Q2 = 0.451)).

First, unsupervised PCA models were established for the data in positive and negative ion modes for NS, CHD, and RA groups. As shown in Figure 1A, NS and RA, and NS and CHD can present a good separation effect. Then, the supervised PLS-DA analysis was able to obtain the specific variables that caused the differences between the groups. As shown in Figures 1B–D, both disease groups show a significant categorical aggregation with the NS group on the scatter plot, indicating that the metabolic pattern of the disease group differs significantly from that of the control group, with significant abnormal changes in endogenous metabolites in the serum of patients with both CHD and RA.

In this study, we used the PLS-DA results to screen the information of substances with the same significant difference (*p* < 0.05) between CHD and RA groups and then screened 21 biomarkers common to CHD and RA. Among them, 10 metabolites showed upregulation in both disease groups, 11 metabolites showed downregulation in both diseases, and bilirubin showed opposite trends in both diseases, as shown in Table 1.

**TABLE 1:** Ion information of disease biomarkers.

No.	Name	Molecular formula	Parent ion	Measured value	Theoretical value	Error (ppm)	RA/NS	CHD/NS
1	Glutaminylglutamic acid	C_10_H_17_N_3_O_6_	M + Na	298.1018	298.101	2.68	0.47	0.45
2	1,2-Dioleoyl-sn-glycero-3-phosphocholine	C_44_H_84_NO_8_P	M + H	786.5996	786.6007	-1.4	0.82	0.79
3	Tryptophyl-Lysine	C_17_H_24_N_4_O_3_	M + H	333.1876	333.1921	-13.51	1.61	2.08
4	Tryptophyl-Phenylalanine	C_20_H_21_N_3_O_3_	M + H	352.1657	352.1656	0.28	0.77	0.76
5	16-α-Hydroxypregnenolone	C_21_H_32_O_3_	M + Na	355.2296	355.2244	14.64	1.81	1.80
6	Citric acid	C_6_H_8_O_7_	M + Na	215.017	215.0162	3.72	0.52	0.51
7	N-gamma-Glutamylglutamine	C_10_H_17_N_3_O_6_	M + H	276.1194	276.119	1.45	0.62	0.62
8	Melanostatin	C_13_H_24_N_4_O_3_	M + H	285.1885	285.1921	-12.62	0.60	0.70
9	CerP (d18:1/16:0)	C_34_H_68_NO_6_P	M + H	618.4779	618.4857	-12.61	0.51	0.62
10	Palmitoylethanolamide	C_18_H_37_NO_2_	M + H	300.2904	300.2897	2.33	1.88	1.66
11	Cer(t18:0/16:0)	C_34_H_69_NO_4_	M + H	556.5297	556.5299	-0.36	2.07	1.46
12	CerP (d18:1/12:0)	C_33_H_68_NO_6_P	M + H	606.4957	606.4863	5.70	0.42	0.57
13	PE (14:0/16:1 (9Z))	C_35_H_68_NO_8_P	M + Na	684.4549	684.4575	-3.8	0.63	0.55
14	Arginylglutamine	C_11_H_22_N_6_O_4_	M + H	303.178	303.1775	1.65	1.56	2.08
15	Bilirubin	C_33_H_36_N_4_O_6_	M + H	585.2663	585.2708	-7.69	0.84	1.31
16	2-Hydroxyhexadecanoylcarnitine	C_25_H_47_NO_4_	M + H	426.3581	426.3578	0.7	1.01	1.20
17	Stearoylcarnitine	C_25_H_49_NO_4_	M + Na	450.3574	450.3554	4.44	1.19	1.28
18	Ureidoisobutyric acid	C_5_H_10_N_2_O_3_	M + H	147.1156	147.0764	-14.96	0.84	0.81
19	13′-Hydroxy-gamma-tocopherol	C_28_H_48_O_3_	M + K	471.3197	471.3235	-8.06	1.42	1.94
20	Linolenelaidic acid	C_18_H_30_O_2_	M + K	317.1901	317.1877	7.57	1.49	1.90
21	Alpha-Linolenic acid	C_18_H_30_O_2_	M + K	317.1901	317.1877	7.57	1.49	1.90

#### 3.1.2 Cluster analysis

**FIGURE 2:**
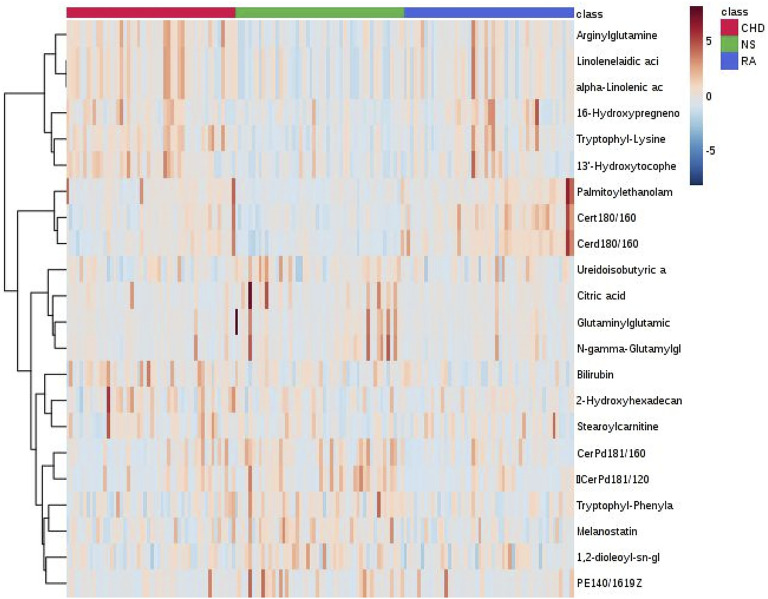
Heat map analysis of common biomarkers for RA-CHD disease.

The Heatmap can more intuitively show the changes in biomarkers in each group. As shown in Figure 2, the colors of these 21 diagnostic biomarkers were more uniform under the same group, whereas the color shades in both the CHD and RA groups were significantly different from those in the NS group, indicating that the contents of these metabolites in the NS group were significantly different from those in the CHD and RA groups, verifying the discriminatory ability of these differential metabolites.

#### 3.1.3 Evaluation of the diagnostic ability of rheumatoid arthritis-coronary heart disease biomarkers

**FIGURE 3:**
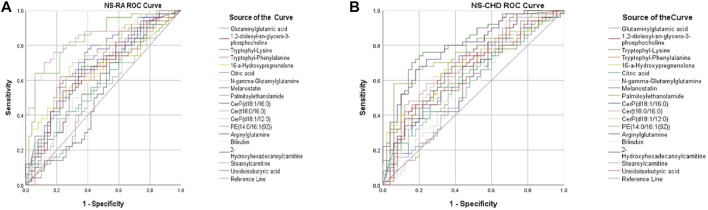
Disease marker ROC curve. **(A)** RA marker ROC curve, **(B)** CHD marker ROC curve).

To further explore whether these markers have diagnostic significance, the ROC curve analysis function in SPSS 17.0 software was used to verify and judge the diagnostic ability of the above-screened substances. The curves in different colors in the figure represent one biomarker each (Figure 3). Through ROC curve analysis, the AUC of 21 markers were obtained in the NS-RA and NS-CHD groups, and the total AUC of 21 markers, with AUC of >0.7 for each substance and their distribution between 0.71 and 0.97 (95% confidence interval), indicating the diagnostic significance of the markers screened in this experiment.

#### 3.1.4 Mate-PA metabolic pathway analysis

**FIGURE 4:**
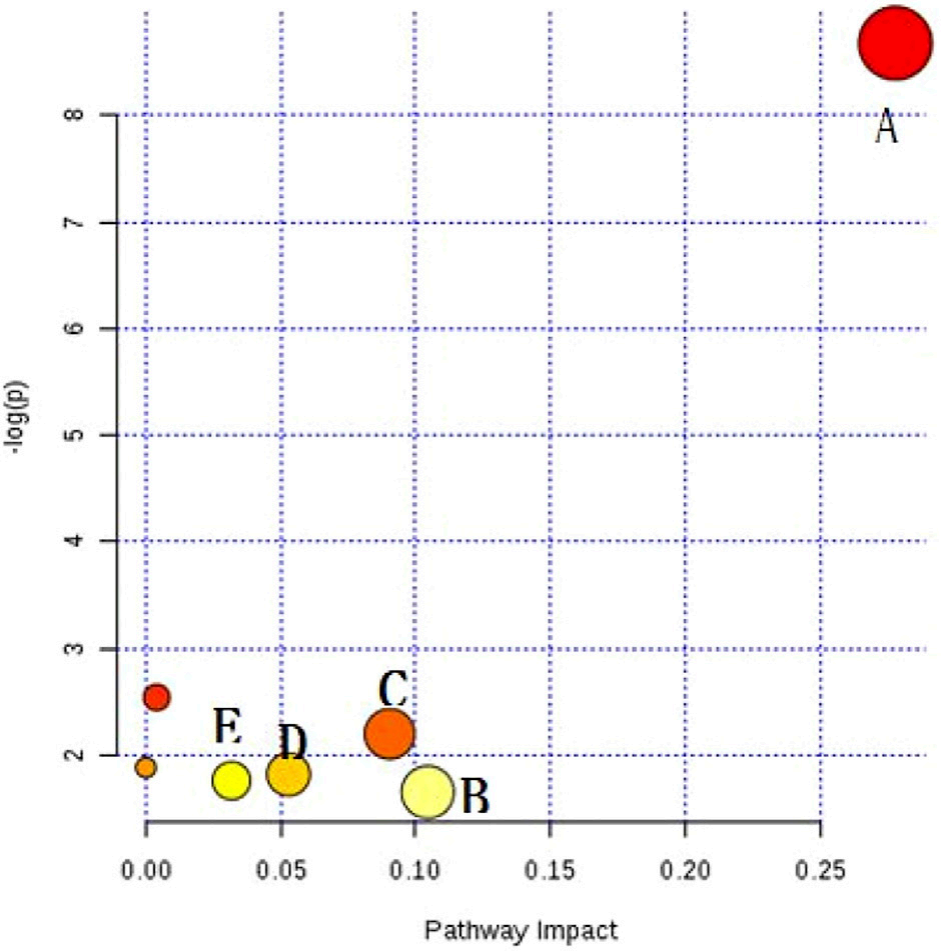
Disease Mateker–PA diagram. **(A)** Sphingolipid metabolism, **(B)** glycosylphosphatidylinositol (GPI)-anchored biosynthesis, **(C)** citrate cycle, **(D)** porphyrin and chlorophyll metabolism, **(E)** glyoxylic acid and dicarboxylic acid ester metabolism.

The resulting biomarkers shared by the two diseases were imported into the meta-PA database, and the markers were subjected to metabolic pathway analysis to infer the details of metabolic disorders caused by CHD and RA. Meta-PA analysis indicates that CHD and RA may affect the above pathways and dysregulate the overall status, with the most pronounced effect on the sphingolipid metabolism pathway (Figure 4).

### 3.2 Network pharmacology analysis results

#### 3.2.1 Establishment of shared target library for coronary heart disease and rheumatoid arthritisdiseases

In this study, the target information of the CTD database was used as the basis, and the TTD, durgbank, and OMIM databases were used to supplement the search of targets related to CHD and RA. A total of 4497 CHD disease and treatment-related targets and 1371 RA disease and treatment-related targets were obtained. The obtained CHD and RA targets were subjected to Venny analysis, and 970 targets were obtained for both diseases.

#### 3.2.2 Construction of PPI network for common disease targets

The 970 shared targets were imported into the STRING database for protein interaction analysis, and an interaction map of 970 proteins with tight interactions was obtained. This map was then imported into Cytoscape 3.6.1 to map the target protein PPI network. In addition, the further filtering condition of degree greater than 10 was used to obtain 480 core targets and 20,037 edges common to both diseases.

#### 3.2.3 Enrichment analysis and visualization of coronary heart disease and rheumatoid arthritis pathways

**FIGURE 5:**
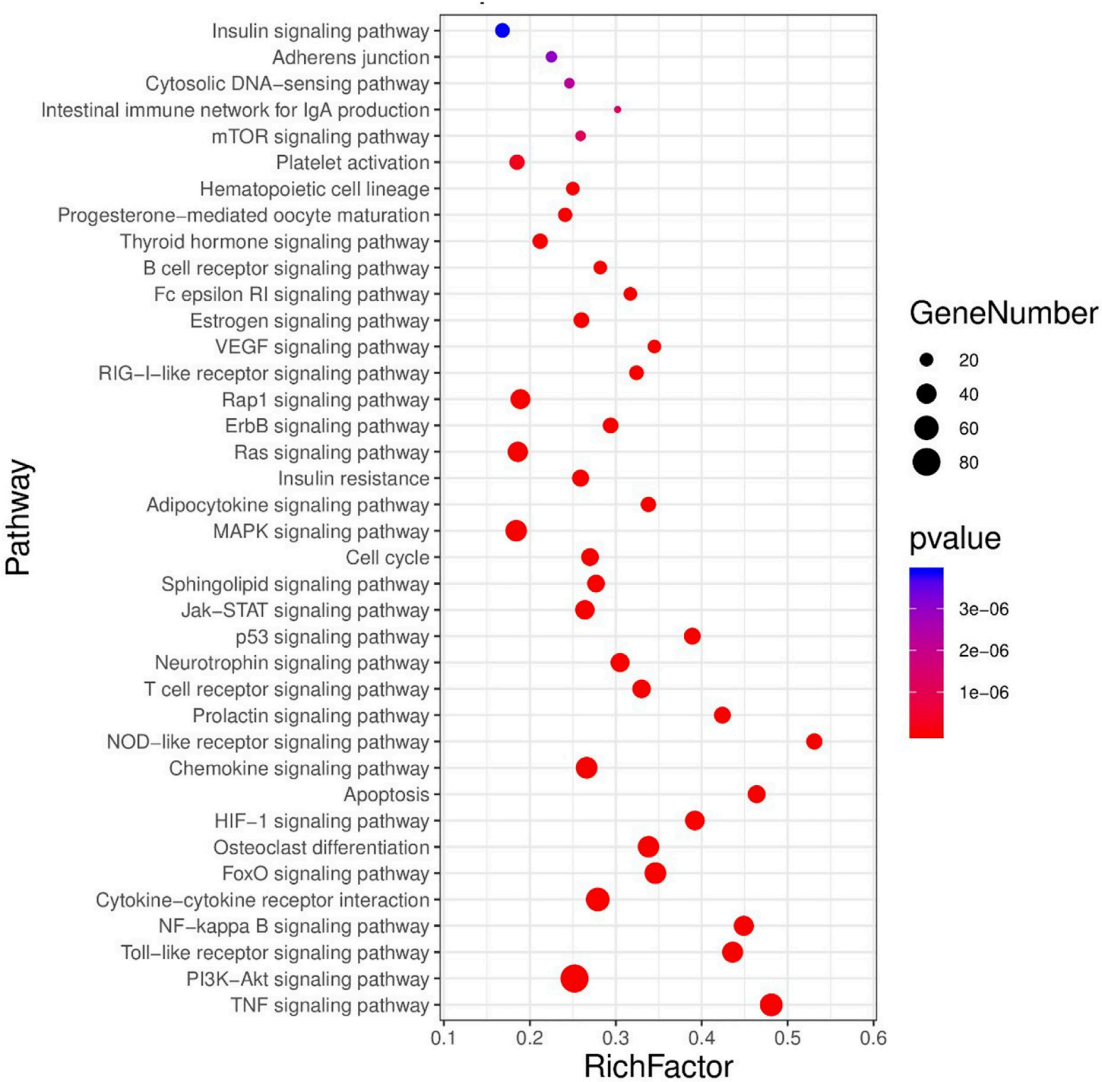
High-level bubble diagram of KEGG enrichment pathway, a common target of CHD and RA.

There were 38 significantly related metabolic pathways (*p* < 0.05, FDR < 0.01). The enriched pathways were visualized by the advanced bubble map function of the OmicShare cloud platform (Figure 5).

Consistent with previous studies on the two diseases, we could realize that the target of comorbidity of the two diseases is mainly concentrated in the inflammatory, immune, and apoptosis signaling pathways. In addition, the above-mentioned key network targets are also significantly enriched in hormone-related pathways, which indicates that there is a certain correlation between the two diseases and the disorder of hormone metabolism. The abnormality of three signal pathways related to sex hormone is consistent with the large proportion of women in patients with two clinical diseases. In addition, the signaling pathways of the two diseases are abnormally enriched in lipid metabolism-related pathways, such as sphingolipid signaling pathway and adipocytokine signaling pathway, suggesting that lipid regulation may be the direction of treatment.

#### 3.2.4 Collection results of main effective components and targets of YiYiFuZi Powder

The active ingredients of the two Chinese herbs in coix seed and Fuzi were screened by the TCMSP website. In this study, combining the drug-like property of ≥0.18 and oral bioavailability of ≥30% as the screening conditions for active ingredients, nine out of 38 chemical components of coix seed and 21 out of 65 chemical components of Fuzi met the criteria (Supplementary Appendix Table S1). The target information was summarized from the TCMSP and Swiss Target Prediction websites, and the common targets were removed to obtain a total of 543 constituent targets of YYFZ powder.

#### 3.2.5 Mapping of YiYiFuZi powder and rheumatoid arthritis-coronary heart disease common targets and PPI network construction

**FIGURE 6:**
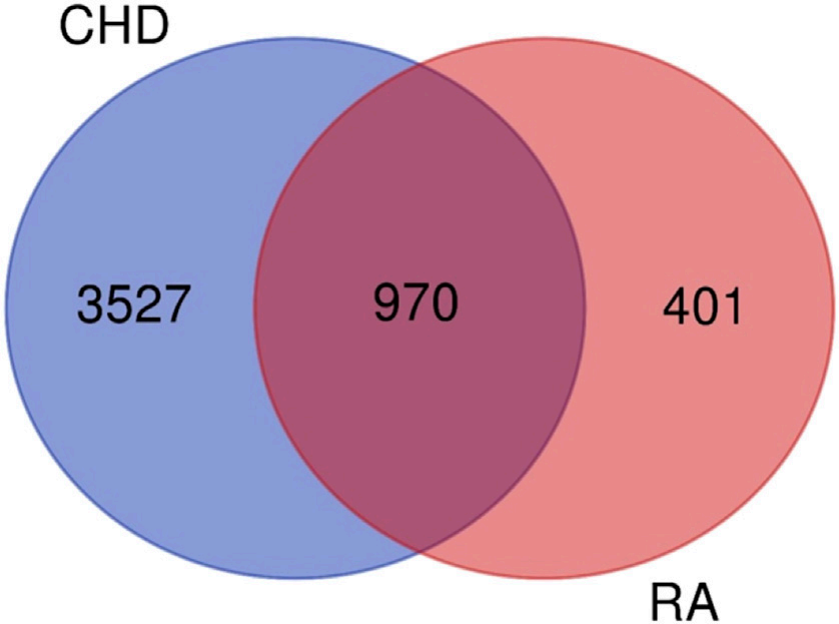
Venn diagram of common target of disease and component target of YiYiFuZi (YYFZ) powder.

**FIGURE 7:**
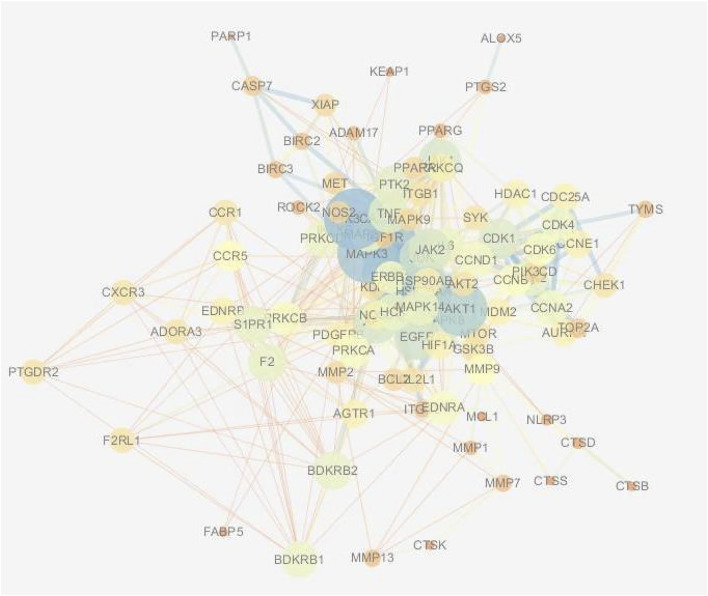
Drug disease common target PPI map.

The results of the Venn enrichment analysis of the main correlated targets of RA-CHD (480) and the targets of YYFZ powder (543) are shown in Figure 6, and 99 targets of YYFZ powder were obtained to modulate the two diseases. The component-target-disease network was constructed by importing the targets of YYFZ powder and the common targets of the two diseases into Cytoscape, and the PPI network was constructed by analyzing the interactions of the common target proteins, and the results are shown in Figure 7.

#### 3.2.6 Biological network KEGG pathway enrichment analysis and visualization results

The results of target pathway correspondence and pathway importance visualization are shown in Figure 5. The results can be seen that its therapeutic effects are mainly from hormonal signaling pathways (progesterone, estrogen, thyroid hormone, etc.), immune, and inflammatory signaling pathways. In conclusion, our results indicate that much of the disease regulation focused on immunoinflammatory and apoptotic signaling pathways. In addition, lipid and hormonal signaling pathways have been found to be important in the regulation of different diseases and different treatments by YYFZ powder.

#### 3.2.7 GO analysis results

**FIGURE 8:**
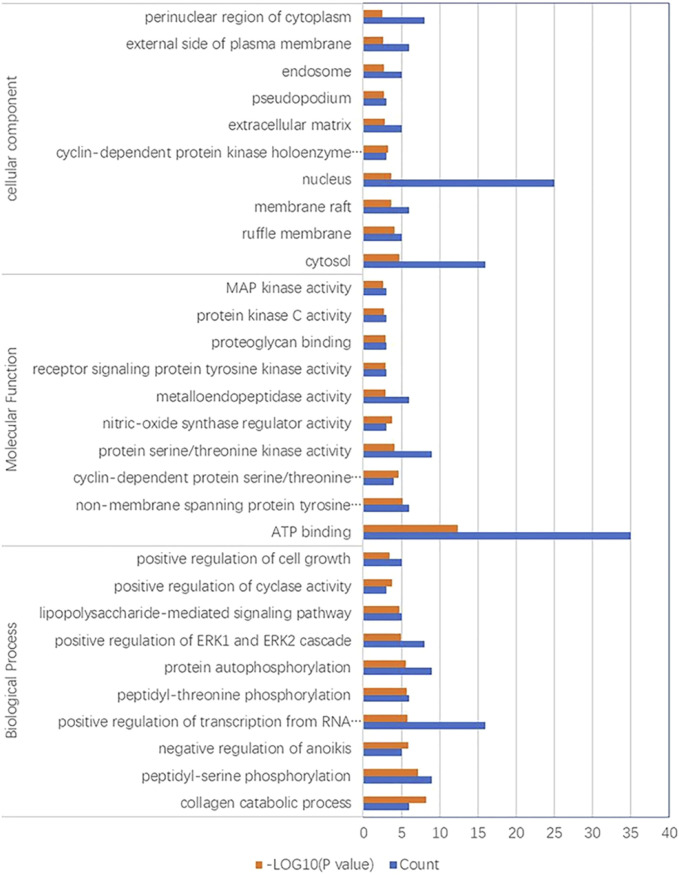
GO function enrichment analysis.

In terms of biological processes, 103 enrichment results were obtained, and the regulatory targets of YYFZ powder that exerted allopathic treatment mainly involved collagen catabolic process, peptidyl serine phosphorylation, and positive regulation of RNA polymerase II promoter transcription. In terms of molecular function, a total of 19 enrichment results were obtained, with a total of 35 of the shared targets enriched in ATP binding function and nontransmembrane protein tyrosine kinase activity. The part of cellular composition involved 14 enrichment results, and target proteins were mainly present in the cytoplasm, nucleus, and cytoplasmic lysate. The top 10 analysis results of GO analysis are shown in Figure 8.

### 3.3 Integration of analysis results

Through the integration of network pharmacology and metabolomics results and the search of related literature, the following conclusions were drawn: Disorders of sphingolipid metabolism in CHD and RA cause activation of downstream signaling pathways such as apoptosis and inflammation, and inhibition of ceramide synthesis may be a key therapeutic target for the cotreatment of CHD and RA. The results of network enrichment analysis of the two disease comorbidities show that the sphingolipid signaling pathway occupies an important position in the two disease comorbidities (Supplementary Appendix Figure S1). In addition, ASM enzymes were enriched in the comorbid targets of the two diseases, and it is speculated that ASM enzymes may be the key targets for the treatment of the two diseases. In addition, the ASM enzyme (gene name: SMPD1), an upstream synthesis target of ceramide located in the sphingolipid signaling pathway, was also enriched in the disease target enrichment in this study.

The literature research revealed that the ASM enzyme occupies an important role in ceramide synthesis, and the association of sphingolipase with the two diseases has been reported, which provides a basis for the metabolomics and network pharmacology experiments derived from “The importance of abnormalities in sphingolipid metabolism and sphingolipid signaling pathways in the comorbidity of the two diseases” and the possibility that ASM enzymes are key targets for the comorbidity of the two diseases provide theoretical support (Chimin et al., 2017; Scheiblich et al., 2017; Hong et al., 2019; Kurz et al., 2019). As a clinical cotreatment drug for CHD and RA, there is a lack of reports on the therapeutic mechanism and pharmacodynamic components of YYFZ powder, and whether it can exert therapeutic effects directly through the regulation of ceramide synthesis is yet to be investigated. To this end, this study was conducted to investigate the active components directly bound by ASM enzymes in YYFZ powder by molecular docking and atomic force microscopy.

### 3.4 Molecular docking validation results

**FIGURE 9:**
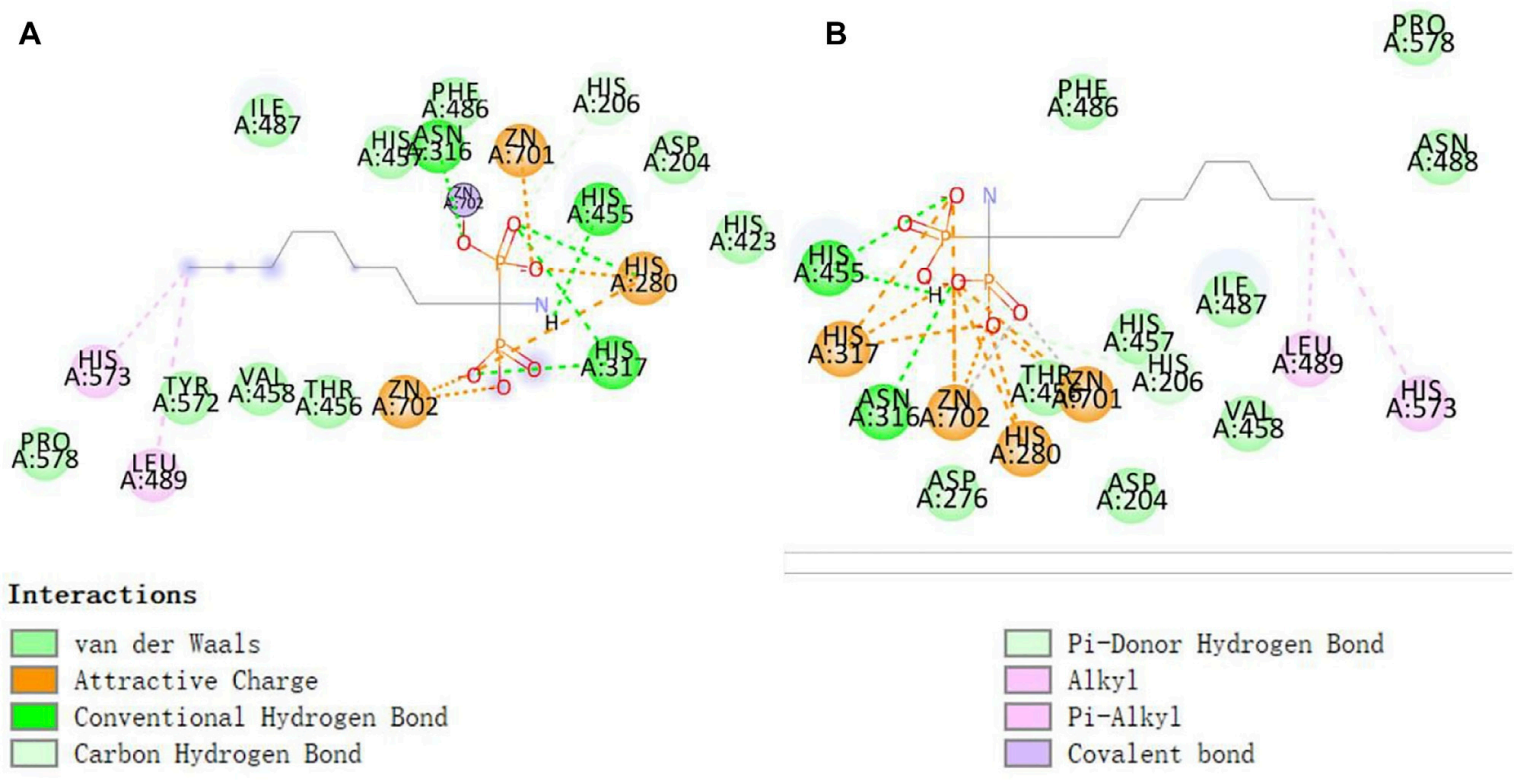
Results of interaction between self-contained ligand **(A)** and protein active cavity before extraction and after redocking **(B)**.

As shown in Figures 9A, B, the conformation of the self-ligand in the ASM active cavity after docking can overlap well with the conformation of the substrate in the original crystal structure with an RMSD of 1.4477 Å < 2.0 Å. This proves that the docking parameters used in this experiment are set reliably and can be used for subsequent virtual screening studies.

#### 3.4.1 CDOCKER score result analysis

According to the analysis of the docking scoring (_CDOCKER-INTERACTION-ENERGY value) results, the main components of YYFZ powder with drug-forming properties can bind well to ASM proteins and enter the protein cavity for reasonable interactions, among which the top three are higenamine (73.2564), β-sitosterol (47.8923), and stigmasterol (46.6821).

#### 3.4.2 Identification of active sites and key amino acid residues in the structure of acid sphingomyelinase enzymes

**FIGURE 10:**
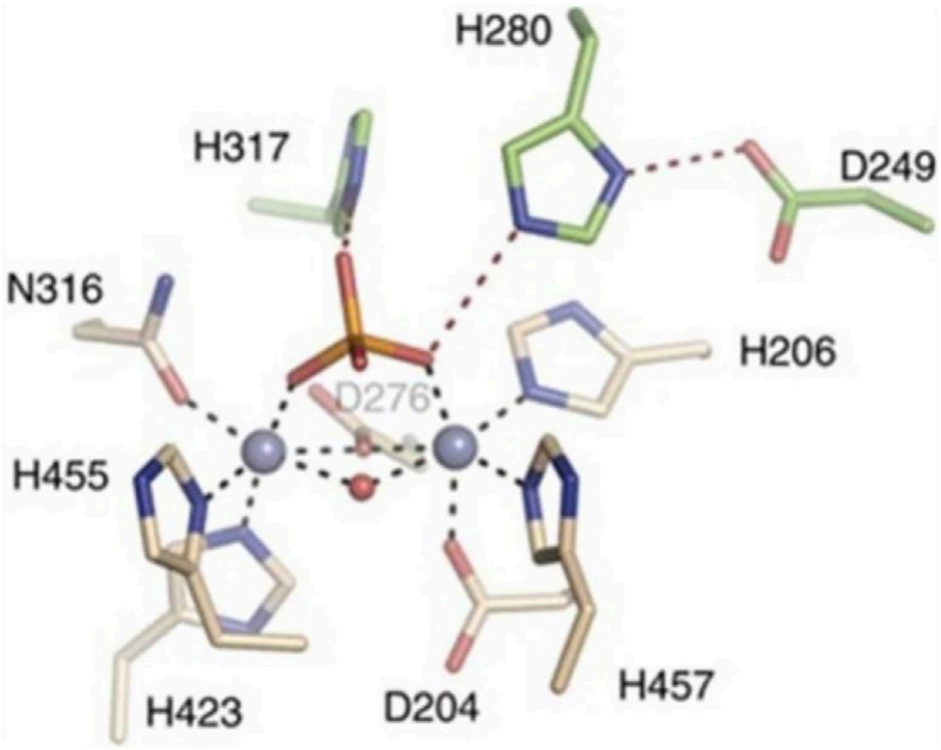
Interaction of zinc ions, amino acid residues, and their own ligands in acid sphingomyelinase (ASM) enzyme structure.

As shown in Figure 10, His317 and His280 are located near the bound phosphate and act as potential proton donors for the ceramide leaving group. It was shown that ASM catalyzes the hydrolysis of sphingolipids through the classical mechanism used by phosphodiesterases, in which nucleophilic attack of zinc-activated water molecules and His280 protonate the ceramide leaving group and release ceramide and phosphorylcholine (Gorelik et al., 2016).

#### 3.4.3 Identification of active sites and key amino acid residues in the structure of Higenamine, β-sitosterol, and stigmasterol

**FIGURE 11:**
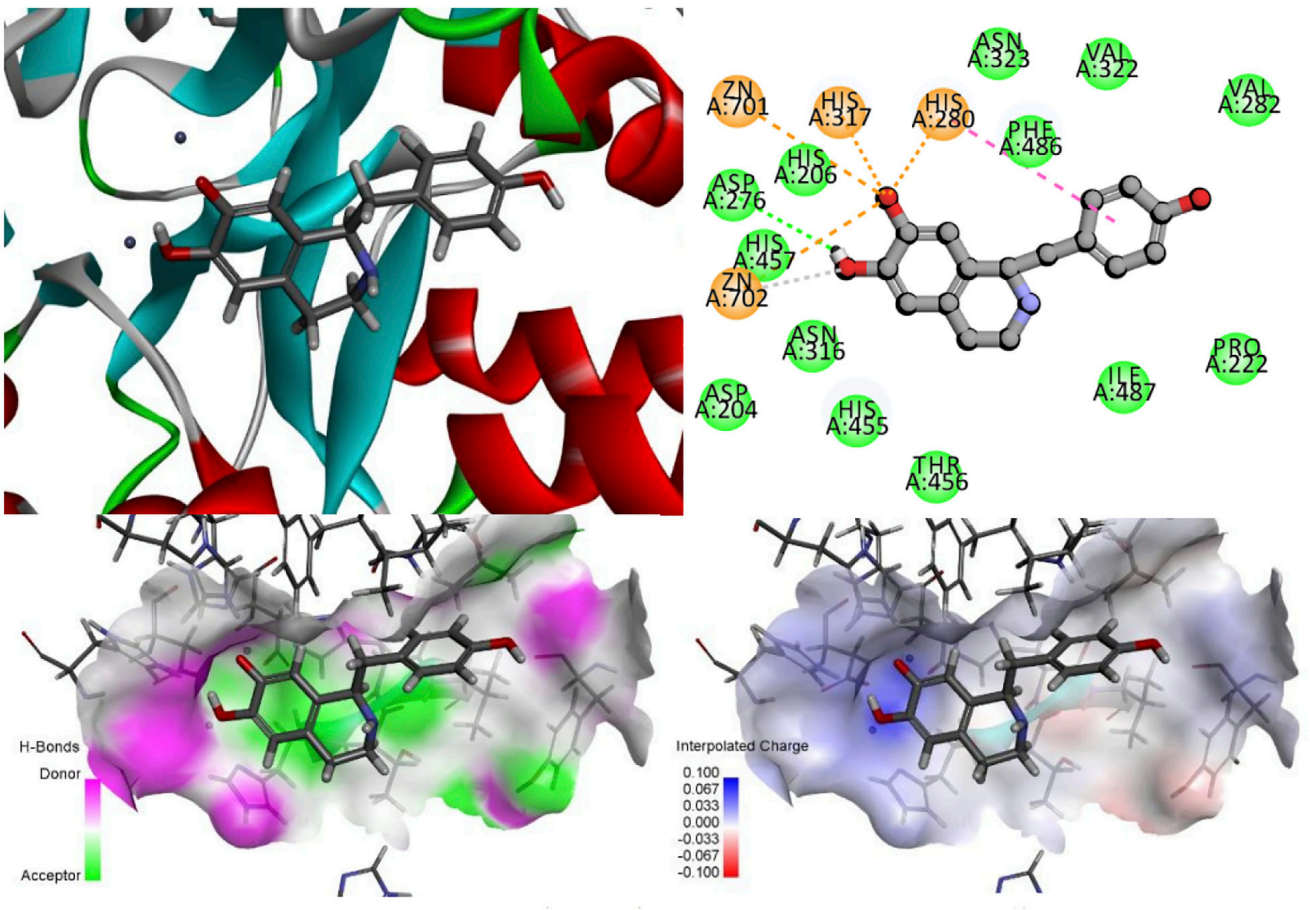
Higenamine interacts with ASM proteins.

The ZN^2+^ ion in the ASM protein structure forms a salt bridge with the oxygen atom in the isoquinoline parent nucleus structure of desmethylaconitine, and the two key amino acids, His317 and His280, in the structure both form electron-absorbing interactions with the oxygen atom; on the other hand, His280 also forms a Pi-Pi T-sharp interaction with the benzene ring in the structure, and the amino acid ASP276 and the hydroxyl group in the structure form hydrogen bonding interactions to provide the stability of the structure (Figure 11).

**FIGURE 12:**
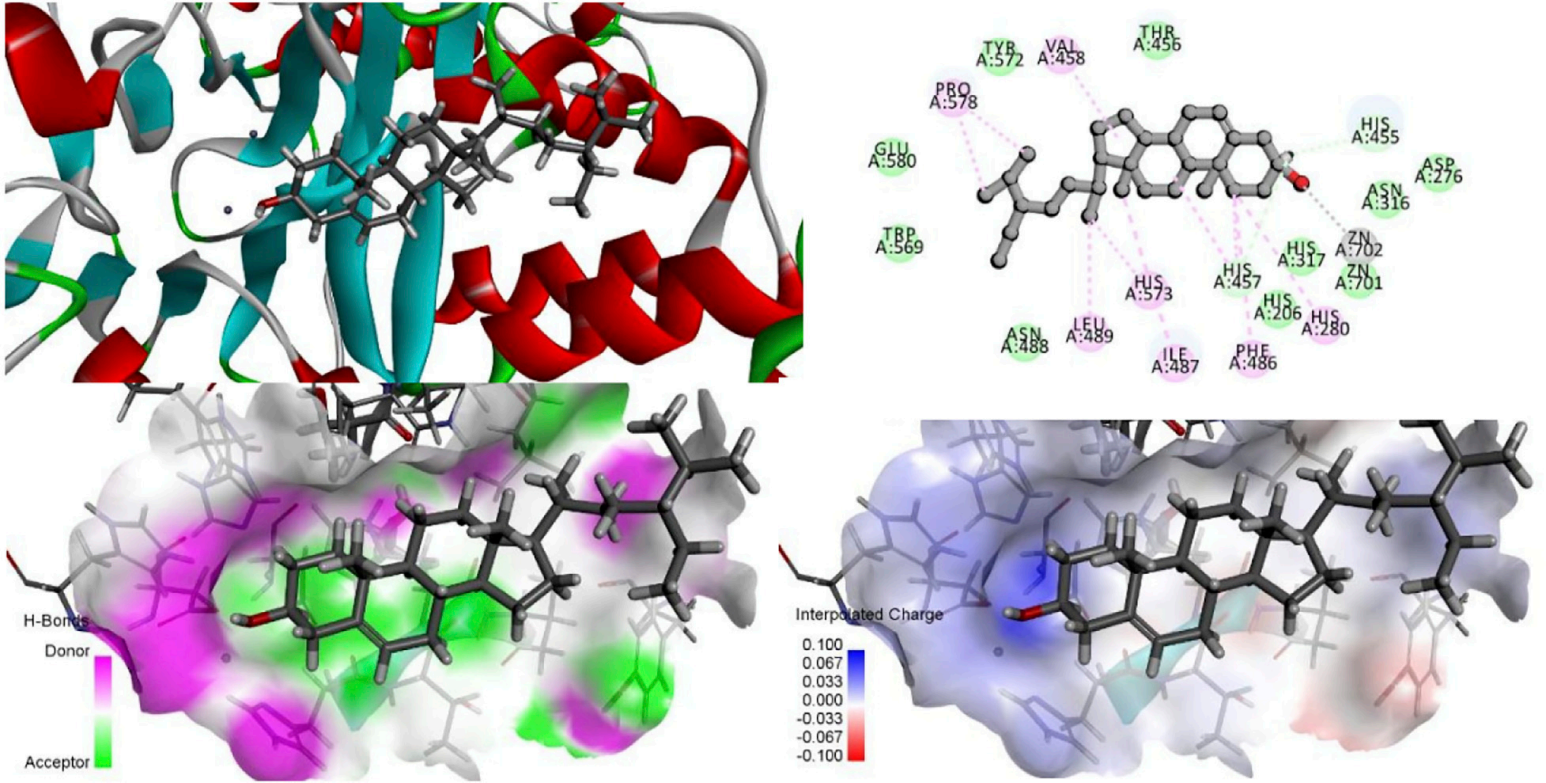
β-sitosterol interacts with ASM proteins.

**FIGURE 13:**
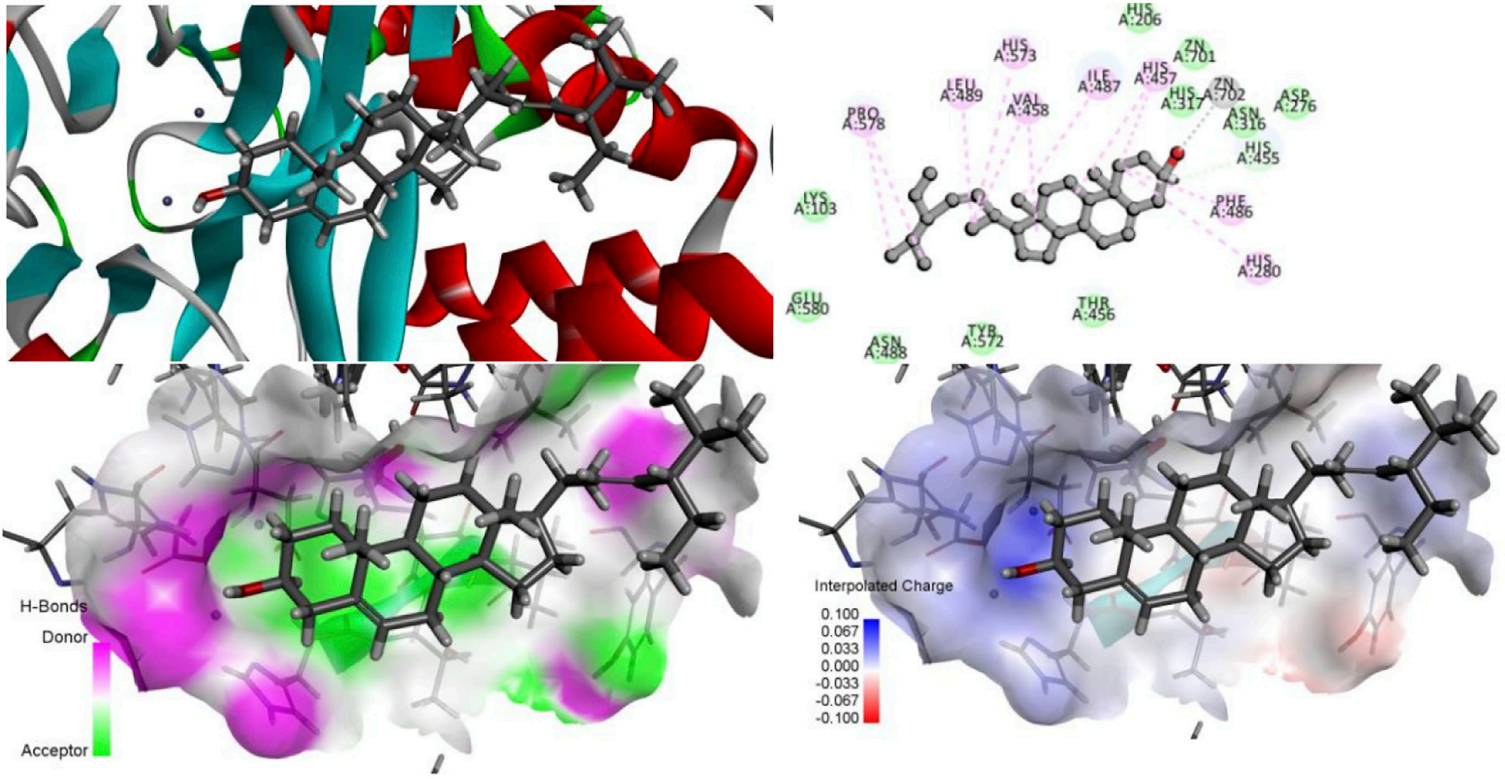
Stigmasterol interacts with ASM proteins.

Since both stigmasterol and β-sitosterol have the mother nucleus of tetracyclic triterpene in their structures and have structural similarity, the same electron-absorbing forces, carbon–hydrogen bonds, and some noncovalent bonds work during the interaction between β-sitosterol (Figure 12), stigmasterol (Figure 13), and ASM protein, and the two ZN^2+^ have strong electron-absorbing as well as van der Waals forces with the oxygen atom in the structure of β-sitosterol and stigmasterol; the two key amino acid residues in the structure of ASM enzyme His280 and His317 form Pi–Alkyl interaction and van der Waals force, respectively. These binding results showed that the stigmasterol was also able to bind better in the protein-active cavity.

### 3.5 Effect of compounds on the surface morphology of acid sphingomyelinase proteins

**FIGURE 14:**
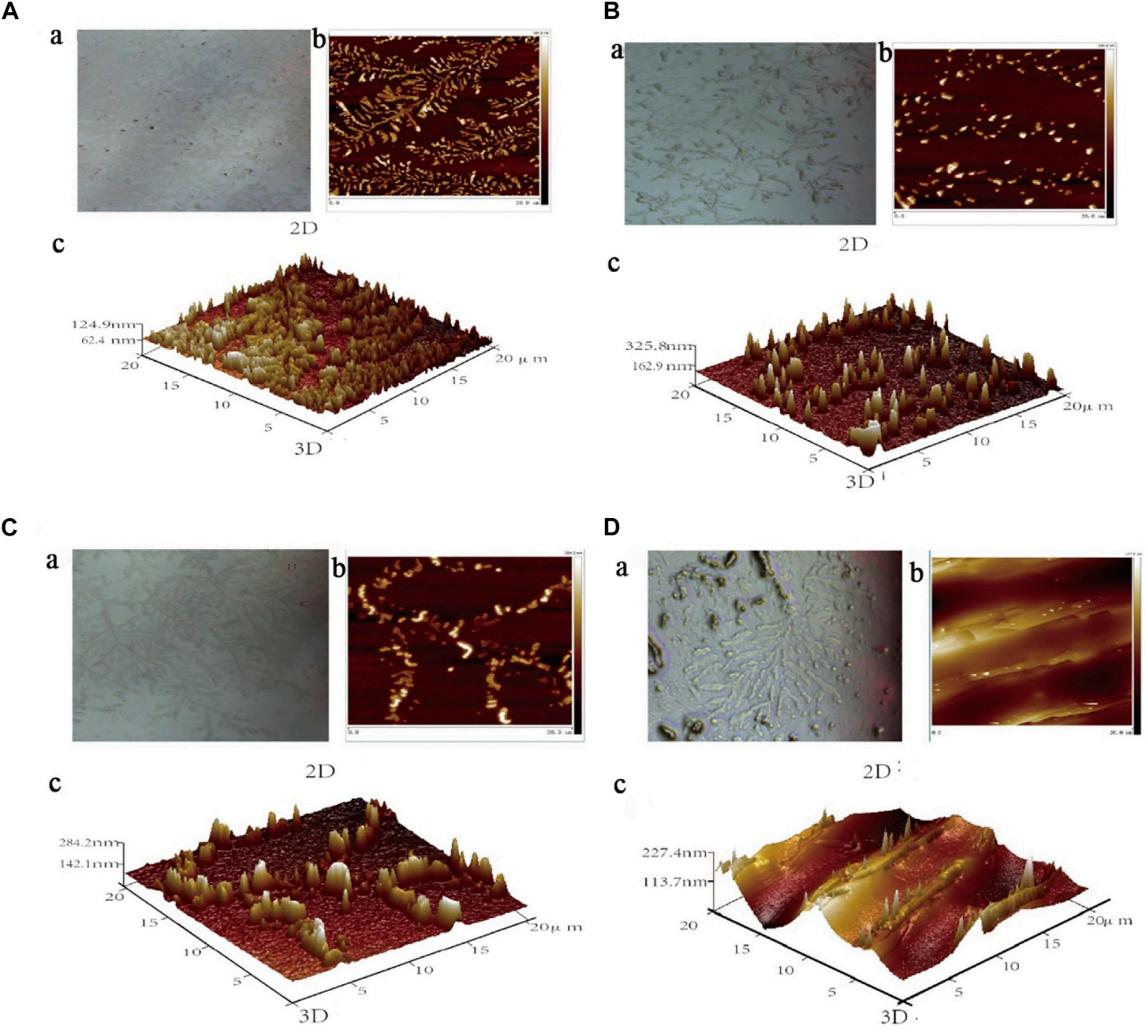
2D and 3D maps of ASM protein **(A)** binding Higenamine **(B)**, β-Sitosterol **(C)**, and Stigmasterol **(D)**.

The results of atomic force microscopy showed that the ASM protein molecules adsorbed on the surface of the quartz sheet were chain-like (Figure 14A) and dispersed before the ASM protein was bound to small molecules, and the height of the ASM protein molecules was 124.9 nm. In addition, the 2D and 3D microscopic imaging showed that the ASM protein changed from a short and rounded, disordered arrangement to a high and stiff, ordered arrangement after the binding interaction with higenamine, β-sitosterol, and stigmasterol.

As shown in Figure 14B, the degree of protein aggregation after the binding of higenamine and protein was the most significant, and the height changed significantly, which was approximately three times the original height. When β-sitosterol interacted with ASM protein, the obvious aggregation of ASM protein into clusters also appeared on the quartz sheet, and its molecular height was 284.2 nm, which was approximately more than twice the original height (Figure 14C). As shown in Figure 14D, and after the binding of stigmasterol with ASM protein, although the protein height change and aggregation phenomenon were not as obvious as in the first two compounds, a certain degree of protein aggregation into clusters also appeared on the quartz sheet, and its molecular height was 227.4 nm, which was approximately twice the original height.

## 4 Discussion

Existing studies have shown that CHD and RA share similar metabolic disorders and intersect in molecular signaling pathways related to inflammation and immunity. To show the comorbidity mechanism of CHD and RA and find the direction of cotreatment in the diagnosis and treatment of the two diseases, this study analyzed the potential cotreatment targets of CHD and RA through network pharmacology technology and conducted the enrichment analysis of the common pathways of the two diseases to explore the comorbidity mechanism of the two diseases. The pathway enrichment results showed that the development of the two diseases is closely related to several biological processes, including inflammatory response (TNF signaling pathway, NF-κB signaling pathway, etc.), immunity (PI3K-Akt signaling pathway, Jak-STAT signaling pathway, Toll-like receptor signaling pathway, etc.), and regulation of vascular endothelial function (estrogen signaling pathway, HIF-1 signaling pathway, VEGF signaling pathway, apoptosis signaling pathway, etc.).

Ceramides are key signaling lipids that regulate the biophysical properties of membranes and are involved in many cellular processes as well as disease states. In a study on the regulation of sphingolipid metabolism in RA rats by Chinese herbal medicine, the Chinese herbal medicine compound IDM exerted antiarthritic effects by inhibiting Cer-mediated COX-2 activation and leading to PEG2 release, suggesting that ceramide plays an important role in RA and coronary atherosclerosis (Qu et al., 2018). Acid sphingomyelinase (ASMase, ASM, SMPD1) is a zinc-dependent phosphodiesterase that catalyzes the conversion of sphingomyelin (SM) to ceramide and phosphorylcholine at the cell membrane or lysosomal membrane. On the other hand, studies have found that the inhibition of ASM activity destabilizes their lysosomes to selectively kill cancer cells, attenuates inflammation associated with cystic fibrosis, reduces atherosclerotic lesions, and alleviates symptoms associated with Alzheimer’s disease (Kurz et al., 2019). These findings suggest that ASM is a key target for the treatment of related diseases, and ASM enzyme activity has been found to be significantly higher in patients with cardiovascular disease, diabetes, or major depression than in normal subjects (Yang et al., 2018). In RA clinical studies, the mean serum S-SMase activity was twofold higher in RA patients than in normal controls (Hanaoka et al., 2017). In addition, the ASM enzyme was analyzed in a mouse model of inflammatory arthritis by ASM knockdown as well as amitriptyline treatment for significant backregulation of joint swelling and proinflammatory cytokine levels in joint inflammation (Beckmann et al., 2017). However, this study did not address the mechanism by which ASM inhibition ameliorates the severity of arthritis, so it is interesting to investigate the mechanism of ASM-ceramide-induced rheumatoid.

The binding strength of small molecules of YYFZ powder cochinchinensis epithelium to target proteins was evaluated using the molecular docking technique. In addition, the compounds were screened based on the scoring results, and the changes in the molecular morphology of target proteins induced by the selected active molecules were revealed via atomic force microscopy. The results showed that the components higenamine, β-sitosterol, and stigmasterol in YYFZ powder had strong interactions with ASM, and the results of AFM showed that higenamine, β-sitosterol, and stigmasterol were able to bind ASM protein at the protein level, affecting its structure and function. The results of this study confirmed the interaction between the active ingredients of the virtual screened drugs and the target proteins by the results of AFM experiments and may be useful for the screening and establishment of small molecule ligand drugs targeting receptor proteins.

## 5 Conclusion

Based on clinical metabolomics combined with bioinformatics technology, this study attempted to characterize the intrinsic material basis of YYFZ powder in the treatment of CHD and RA. In addition, we attempted to explore the pharmacodynamic components of YYFZ powder in the treatment of different diseases from both direct and indirect perspectives. On the one hand, through the untargeted metabolomic analysis of serum samples from the two diseases and the NS group, the differential metabolic markers of CHD and RA comorbidity and the mechanism of comorbid metabolic disorders were obtained. This analysis revealed that the metabolic disorders of the two diseases mainly focused on sphingolipid metabolism, inflammation, oxidative stress metabolic pathways, and the abnormalities of ceramide played an important role in the two diseases. On the other hand, YYFZ powder is enriched in its component regulatory targets for the cotreatment of the two diseases through network pharmacology to elaborate its indirect mechanism for the treatment of the disease. The codisease targets of the two diseases mainly focus on inflammatory immunity, hormones, and lipid signaling pathways.

Metabolomics and the analysis results of CHD and RA disease network modules were integrated and analyzed, and the abnormality of ceramide was judged to play an important role in the two diseases using the results of metabolomics. Through network pharmacology, the disease was enriched in common targets and enriched in ASM enzymes. At present, there are relevant reports on the research of ASM enzyme-amitriptyline in RA and atherosclerosis. In summary, it is inferred that ASM protein occupies an important position in the two diseases, and screening and discovering direct inhibitors of ASM enzyme may be a breakthrough in the treatment of the two diseases. The results of molecular docking and atomic force microscopy showed that the binding strength of higenamine, β-sitosterol, and stigmasterol to the ASM enzyme was high, and the binding conformation was reasonable, which might be the direct pharmacodynamic components of YYFZ powder to exert therapeutic effects.

## Data Availability

The original contributions presented in the study are included in the article/Supplementary Material further inquiries can be directed to the corresponding authors.
