# Supplementary material for: Study on the potential mechanism of the active components in YiYiFuZi powder in homotherapy for hetropathy of coronary heart disease and rheumatoid arthritis
Source: Front Chem. 2022 Aug 9;10:926950. doi: 10.3389/fchem.2022.926950 (PMC9395646; doi:10.3389/fchem.2022.926950)
Supplement: Supplementary file 1 [file DataSheet1.docx]

1. Instruments and Materials

1.1 Experimental instruments

Table 1 Experimental instrument

| Name | Manufacturers |
| --- | --- |
| Frozen high-speed centrifuge | Beckman Corporation, USA |
| Electronic Balance | Shanghai OHAUS Instruments Co. |
| Quick Mixer | Jiangyan Xinkang Medical Equipment Co. |
| UPLC-Q-TOF/MS | Waters Corporation, USA |

1.2 Drugs and reagents

Table 2 Drugs and reagents

| Name | Manufacturers |
| --- | --- |
| Acetonitrile | Merck, Germany |
| Methanol | CNW Germany |
| Physiological saline | Shandong Qidu Pharmaceutical Co. |
| Coix seed | Hunan Yuxingtang Chinese Medicine Technology Co. |
| Fuzi | Hebei Meiwei Pharmaceutical Co. |

1. Methods
   1. Preparation of aqueous decoction of YiYiFuZi powder

The samples were weighed precisely in the ratio of 5:3, placed in a suitable round bottom flask, soaked in 10 times the amount of water for 45 min and refluxed at direct heat for 1 h. After the first reflux, the filtrate was filtered through 3 layers of gauze and set aside; the remaining residue was added with 8 times the amount of water and the second reflux was started for 45 min. After reflux, the residue was filtered off and the two filtrates were combined and concentrated to a viscous(Calculated by crude drug, the concentration is 1 g/ml). The samples were stored in a refrigerator at 4°C and kept in reserve.

- 1. Sample pretreatment

Add 100 μL of methanol to 200 μL of decoction of YiYiFuZi powder, vortex and mix well, centrifuge at 4°C, 13200 *g for 15 min. Remove the supernatant and blow dry with nitrogen, then add methanol and vortex for 1 min, centrifuge at 4°C, 13200 *g for 15 min, and remove the supernatant for subsequent analysis.

- 1. Liquid phase conditions

A Waters ACQUITY UPLC BEH C18 column (2.1 mm×100 mm, 1.7 μm) was used with a column temperature set at 35°C, a flow rate of 0.3 mL/min, and an injection volume of 10 μL. The mobile phase A (0.1% formic acid aqueous solution)-B (0.1% formic acid acetonitrile solution) was used with a gradient elution (Table 3).

Table 3 Elution conditions

| Time(min) | A(%) | B(%) |
| --- | --- | --- |
| 0 | 99 | 1 |
| 1 | 99 | 1 |
| 2 | 95 | 5 |
| 5 | 85 | 15 |
| 10 | 70 | 30 |
| 15 | 50 | 50 |
| 20 | 30 | 70 |
| 24 | 1 | 99 |

- 1. Mass Spectrometry Conditions

An electrospray ionization source (ESI source) was used for the analysis in positive ionization mode, with high-purity liquid nitrogen as an auxiliary spray ionization and desolventizing gas, with a mass spectrometry scan range of m/z 50-1000; capillary voltage 3.0 kV; desiccator temperature 325°C; drying gas flow rate 10 mL/min; desolventizing gas flow rate (N2) 600 L/h; ion source temperature 120 °C; desolvent gas temperature 350 °C; cone gas flow rate 50 L/h, scan range 50-2000.

- 1. Data processing

The UPLC-Q-TOF/MS data of the aqueous decoction of Coix lacryma and serum samples were processed by MassLynx V 4.1 software (Waters, Manchester, UK) for peak detection and extraction. The compounds were identified by ion fragmentation by comparison with the information on the chemical composition of Coix lacryma seeds obtained through literature research.

1. Results and Discussion

In this study, the chemical constituents in the aqueous decoction of Coix lacryma seeds were confirmed by UPLC-Q-TOF-MS technique, and finally 51 in vitro components were identified, of which 42 were alkaloids in YYFZ, one was fatty acid in Coix seeds, two were sterols in Coix seeds, three compounds in Coix lacryma seeds and the remaining three were unknown components with no specific configuration(Table4).

Tab. 5-4 In vitro chemical constituents of aconite dispersion of YYFZ powder

| No. | Rt(min) | Name | Molecular Fomular | | Summation mode | Measurement | Theoretical value | ppm | Fragments | Resource |
| --- | --- | --- | --- | --- | --- | --- | --- | --- | --- | --- |
| 1 | 1.87 | Unknown compound 1 | | C_17_H_33_NO | M+H | 268.2655 | 268.264 | 5.59 | 136.0639,240.9886 |  |
| 2 | 2.86 | Phenylalanine | | C_9_H_11_NO_2_ | M+H | 166.0872 | 166.0868 | 2.41 | 120.0839 | Fuzi |
| 3 | 3.2 | karakolidine | | C_22_H_35_NO_5_ | M+H | 394.262 | 394.2593 | 6.85 | 376.2509,358.2350,340.2399 | Fuzi |
| 4 | 3.34 | chuanfumine | | C_22_H_35_NO_5_ | M+H | 394.2619 | 394.2593 | 6.6 | 376.2521,358.2369,340.2286,322.2157 | Fuzi |
| 5 | 4.03 | senbusine B | | C_23_H_37_NO_6_ | M+H | 424.2711 | 424.2699 | 2.83 | 378.2665,374.2350，406.2613,388.2541 | Fuzi |
| 6 | 4.18 | Hydroxycardiopetaline | | C_22_H_33_NO_4_ | M+H | 364.2511 | 364.2482 | 7.96 | 346.2406,310.1815,300.1789 | Fuzi |
| 7 | 4.27 | mesaconine | | C_26_H_41_NO_6_ | M+Na | 486.2832 | 486.2832 | 0 | 468.2638,436.2386,422.2231 | Fuzi |
| 8 | 4.4 | senbusine A | | C_23_H_37_NO_6_ | M+H | 424.2733 | 424.2699 | 8.01 | 378.2680,374.2308,406.2650,388.2527 | Fuzi |
| 9 | 4.54 | isotalatizidine | | C_23_H_37_NO_5_ | M+H | 408.2767 | 408.275 | 4.16 | 390.2704,358.2439,340.2281,372.2582 | Fuzi |
| 10 | 4.73 | songorine | | C_22_H_31_NO_3_ | M+H | 358.2406 | 358.2382 | 6.7 | 340.2328,322.2195,312.2015 | Fuzi |
| 11 | 4.80 | sczukidine | | C_22_H_31_NO_3_ | M+H | 358.2406 | 358.2382 | 6.7 | 330.2113 | Fuzi |
| 12 | 4.9 | hetisine | | C_20_H_27_NO_3_ | M+H | 330.2083 | 330.2069 | 4.24 | 312.2 | Fuzi |
| 13 | 5.07 | 3-Epiignavinol | | C_20_H_27_NO_4_ | M+H | 346.2042 | 346.2018 | 6.93 | 328.1954,310.1868,300.1989 | Fuzi |
| 14 | 5.26 | Higenamine | | C_16_H_17_NO_3_ | M+H | 272.2133 | 272.2152 | 6.97 |  | Fuzi |
| 15 | 5.34 | contorsine | | C_27_H_35_NO_6_ | M+H | 470.2587 | 470.2543 | 9.36 | 442.1893 | Fuzi |
| 16 | 5.41 | fuziline | | C_24_H_39_NO_7_ | M+H | 454.2839 | 454.2805 | 7.84 | 436.2736,418.2563,404.2474,422.2447 | Fuzi |
| 17 | 5.65 | neoline | | C_24_H_39_NO_6_ | M+H，Na | 438.2883 | 438.2856 | 6.16 | 420.2792,402.2865,388.2472,356.2260,370.2406,460.2522 | Fuzi |
| 18 | 5.94 | sczukitine | | C_28_H_37_NO_6_ | M+H | 484.2712 | 484.2699 | 2.68 | 424.2282 | Fuzi |
| 19 | 6.19 | Talatisamine or Isotalatizamine | | C_24_H_35_NO_5_ | M+H | 422.2928 | 422.2903 | 5.92 | 390.2671,358.2357,372.2466,340.2319 | Fuzi |
| 20 | 6.23 | GuanfuBaseC | | C_22_H_33_NO_2_ | M+H | 344.2605 | 344.259 | 4.35 | 326.2459,308.1971 | Fuzi |
| 21 | 6.81 | 14-acetylneoline | | C_26_H_41_NO_7_ | M+H | 480.2944 | 480.2961 | -3.54 | 462.2919,430.2480,420.2716 | Fuzi |
| 22 | 7.17 | 11-Acetylhetisine | | C_22_H_29_NO_4_ | M+H | 372.2183 | 372.2175 | 2.15 | 312.1965,294.1839 | Fuzi |
| 23 | 7.55 | 14-Benzoyl-10-OH-mesaconine | | C_31_H_43_NO_11_ | M+H | 606.2936 | 606.2914 | 3.63 | 556.2588,588.2809，574.2690 | Fuzi |
| 24 | 8.26 | 14-Benzoyl-10-OH-aconine | | C_32_H_45_NO_11_ | M+H | 620.3064 | 620.3071 | -1.13 | 570.2668,538.2508,602.2934,556.2548 | Fuzi |
| 25 | 8.7 | benzoylmesaconine | | C_31_H_43_NO_10_ | M+H | 590.2976 | 590.2965 | 1.86 | 572.2874,558.2695,540.2589,508.2615 | Fuzi |
| 26 | 9.18 | carmichaenine B | | C_31_H_41_NO_7_ | M+H | 540.298 | 540.2961 | 3.52 | 522.2883,462.2645 | Fuzi |
| 27 | 9.34 | benzoylaconine | | C_32_H_45_NO_10_ | M+H | 604.3146 | 604.3122 | 3.97 | 586.3162,572.2899,554.2792,508.2390 | Fuzi |
| 28 | 9.71 | benzoylhypaconine | | C_31_H_43_NO_9_ | M+H | 574.3037 | 574.3016 | 3.66 | 542.2789,510.2526,524.2595,478.2236 | Fuzi |
| 29 | 10.15 | Dehydrated 14-benzoylmesaconine | | C_31_H_41_NO_9_ | M+H | 572.2877 | 572.286 | 2.97 | 498.3019,522.2542,540.2508，554.2978 | Fuzi |
| 30 | 10.25 | 14-Benzoyl-3,13-deoxyaconine | | C_31_H_43_NO_8_ | M+H | 558.3092 | 558.3067 | 4.48 | 526.2814,508.2722 | Fuzi |
| 31 | 10.3 | 14-Benzoyldeoxyhypaconine | | C_31_H_43_NO_8_ | M+H | 558.3087 | 558.3067 | 3.58 | 526.3065,508.2749,496.2366 | Fuzi |
| 32 | 10.37 | benzoyldeoxyaconine | | C_32_H_45_NO_9_ | M+H | 588.3201 | 588.3173 | 4.76 | 556.2944,542.3130,524.2689 | Fuzi |
| 33 | 10.86 | indaconitine | | C_34_H_48_NO_10_ | M+H | 630.3142 | 630.3278 | -0.21 | 584.4150,602.2192 | Fuzi |
| 34 | 10.98 | 14-Benzoylnoeline | | C_31_H_43_NO_7_ | M+H | 542.3135 | 542.3118 | 3.14 | 526.2814，510.2882,492.2661, | Fuzi |
| 35 | 11.01 | Dehydrated 14-benzoylhypaconine | | C_31_H_41_NO_8_ | M+H | 556.2955 | 556.291 | 8.09 | 524.2697,492.2440 | Fuzi |
| 36 | 11.31 | Dehydrated 14-benzoyldeoxyaconitine | | C_32_H_43_NO_8_ | M+H | 570.3093 | 570.3067 | 4.56 | 538.2833,524.2723,506.2421 | Fuzi |
| 37 | 11.45 | dehydrated-benzoylhypaconine | | C_31_H_41_NO_8_ | M+H | 556.2933 | 556.291 | 4.14 | 538.2850,524.2689,506.2559 | Fuzi |
| 38 | 11.69 | 14-O-anisoylneoline | | C_32_H_45_NO_8_ | M+H | 572.3242 | 572.3223 | 3.32 | 540.3012 | Fuzi |
| 39 | 11.7 | Unknown compound 2 | | C_31_H_41_NO_9_ | M+H | 572.2842 | 572.286 | -3.15 | 540.3008,522.2825,554.2711 | Fuzi |
| 40 | 11.87 | hypaconitine | | C_33_H_45_NO_10_ | M+H | 616.3156 | 616.3122 | 5.52 | 556.2977,524.2700,584.2875,496.2675 | Fuzi |
| 41 | 12.02 | 13-deoxybenzoylhypaconine | | C_33_H_45_NO_9_ | M+H | 600.3173 | 600.3167 | 1.00 | 568.3304,540.2985,508.2685,480.2726 | Fuzi |
| 42 | 12.18 | Foreaconitine | | C_34_H_45_NO_10_ | M+H | 628.3131 | 628.3122 | 1.43 | 568.2952,536.2598 | Fuzi |
| 43 | 12.28 | 8-acetyl-14-benzoyltaltisamine | | C_33_H_45_NO_7_ | M+H | 568.3219 | 568.3274 | 9.68 | 550.3324 | Fuzi |
| 44 | 12.34 | Unknown compound 3 | | C_39_H_41_NO_11_ | M+H | 700.2726 | 700.2758 | -4.57 | 640.2588,654.3068 |  |
| 45 | 12.62 | deoxyaconitine | | C_34_H_47_NO_10_ | M+H | 630.3251 | 630.3278 | -4.28 | 598.3298,570.3073,538.2827,510.2811 | Fuzi |
| 46 | 12.73 | 9,12-Octadecadienoic acid methyl ester or Methyl linoleate | | C_19_H_34_O_2_ | M+H | 295.2631 | 295.2637 | -2.03 |  | Coix seed |
| 47 | 13.07 | Unknown compound 4 | | C_39_H_41_NO_10_ | M+H | 684.2769 | 684.2809 | -5.85 | 624.2585,592.2183 |  |
| 48 | 15.58 | Stigmasterol | | C_29_H_48_O | M+H | 413.7024 | 413.7007 | -6.88 |  | Coix seed |
| 49 | 16.22 | sitosterol | | C_29_H_50_O | M+H | 415.39 | 415.394 | -9.63 | 397.2036 | Coix seed |
| 50 | 19.64 | Unknown compound 5 | | C_18_H_30_O_2_ | M+H | 279.2334 | 279.2324 | 3.58 | 361.0439,217.0406 |  |
| 51 | 25.66 | Unknown compound 6 | | C_32_H_42_NO_6_ | M+H | 537.3059 | 537.309 | -5.77 | 519.2563 |  |

4.1 Identification of alkaloid components

Alkaloid components are the main constituents of Fuzi and are also the main pharmacodynamic active ingredients. In this study, the alkaloids were detected by UPLC-Q-TOF/MS on YYFZ powder and found that alkaloids accounted for the majority of the components in the aqueous decoction. The literature information and database information were queried to summarize the structural analysis of the following alkaloid components. Compound 3 was used as an example for identification.

Compound 3 was detected in the primary spectrum with its excimer ion peak *m/z* 394.2620 [M+H]^+^, and the secondary spectrum showed characteristic fragment ion peaks *m/z* 376.2509 [M+H-H_2_O]^+^ and *m/z* 358.2530 [M+H-2H_2_O]^+^, m/z 3540.2399 [M+H-3H_2_O]^+^. Combined with information from the literature it is presumed to be karakolidine[1].

4.2 Identification of other types of ingredients

Compound 2 has an excimer ion peak of *m/z* 166.0972 [M+H]^+^ in the primary mass spectrum and a characteristic fragment ion peak of *m/z* 120.0839 [M+H-HCOOH]^+^ in the secondary mass spectrum, which is presumed to be Phenylalanine when combined with information from the literature [2]. It is the amino acid in Phenylalanine.

The excimer ion peak of the primary mass spectrometry of compound 45 was *m/z* 295.2631 [M+H]^+^, which could not be confirmed based on the secondary fragment ions due to the lack of cleavage information, and was tentatively presumed to be 9,12-Octadecadienoic acid methyl ester or Methyl linoleate [3], which is a fatty acid component of Coix seed .

Compound 48 has an excimer ion peak of *m/z* 415.3900 [M+H]^+^ in the primary mass spectrum and a characteristic fragment ion peak of *m/z* 397.2036 [M+H-H_2_O]^+^ in the secondary mass spectrum, which is presumed to be sitosterol [3] based on the information from the literature. It is a sterol component of Coix seed.

Reference:

1. Liang Y , Guanu Yan, Jianmin Wu, et al. Qualitative and quantitative analysis of lipo-alkaloids and fatty acids in Aconitum carmichaelii using LC-MS and GC-MS. Phytochemical Analysis Pca, 2018, 29(4): 398−405.
2. Shi Y, Zhao Y, Qian J, et al. Aconitum Diterpenoid Alkaloid Profiling to Distinguish between the Official Traditional Chinese Medicine (TCM) Fuzi and Adulterant Species Using LC-qToF-MS with Chemometrics. Journal of Natural Products, 2021, 84(3).
3. Hl A, Yz A, Ji YB, et al. A comprehensive quality evaluation of Fuzi and its processed product through integration of UPLC-QTOF/MS combined MS/MS-based mass spectral molecular networking with multivariate statistical analysis and HPLC-MS/MS. Journal of Ethnopharmacology, 2021, 266： 113455.

**Appendix**

**Table S1** Structural formula of main chemical components in YYFZ powder

|  | **Molecule Name** | **OB (%)** | **DL** | **Resource** | **Chemical** |
| --- | --- | --- | --- | --- | --- |
| 1 | Neokadsuranic acid B | 43.1 | 0.85 | Fuzi |  |
| 2 | Carnosifloside I_qt | 38.16 | 0.8 | Fuzi |  |
| 3 | sitosterol | 36.91 | 0.75 | Fuzi |  |
| 4 | karakoline | 51.73 | 0.73 | Fuzi |  |
| 5 | isotalatizidine | 50.82 | 0.73 | Fuzi |  |
| 6 | 6-Demethyldesoline | 51.87 | 0.66 | Fuzi |  |
| 7 | benzoylnapelline | 34.06 | 0.53 | Fuzi |  |
| 8 | 2,7-Dideacetyl-2,7-dibenzoyl-taxayunnanine F | 39.43 | 0.38 | Fuzi |  |
| 9 | Deltoin | 46.69 | 0.37 | Fuzi |  |
| 10 | Karanjin | 69.56 | 0.34 | Fuzi |  |
| 11 | Deoxyandrographolide | 56.3 | 0.31 | Fuzi |  |
| 12 | Delphin_qt | 57.76 | 0.28 | Fuzi |  |
| 13 | hypaconitine | 31.39 | 0.26 | Fuzi |  |
| 14 | ignavine | 84.08 | 0.25 | Fuzi |  |
| 15 | deoxyaconitine | 30.96 | 0.24 | Fuzi |  |
| 16 | (3R,8S,9R,10R,13R,14S,17R) | 41.52 | 0.22 | Fuzi |  |
| 17 | (R)-Norcoclaurine（Higenamine） | 82.54 | 0.21 | Fuzi |  |
| 18 | 11,14-eicosadienoic acid | 39.99 | 0.2 | Fuzi |  |
| 19 | Jesaconitine | 33.41 | 0.19 | Fuzi |  |
| 20 | Demethyldelavaine A | 34.52 | 0.18 | Fuzi |  |
| 21 | Demethyldelavaine B | 34.52 | 0.18 | Fuzi |  |
| 22 | Sitosterol alpha1 | 43.28 | 0.78 | Coix seed |  |
| 23 | Stigmasterol | 43.83 | 0.76 | Coix seed |  |
| 24 | β-sitosterol | 36.91 | 0.75 | Coix seed |  |
| 25 | CLR | 37.87 | 0.68 | Coix seed |  |
| 26 | Coixenolide | 32.4 | 0.43 | Coix seed |  |
| 27 | (6Z,10E,14E,18E)-2,6,10,15,19,23-hexamethyltetracosa-2,6,10,14,18,22-hexaene | 33.55 | 0.42 | Coix seed |  |
| 28 | [(2R)-2,3-dihydroxypropyl] (Z)-octadec-9-enoate | 34.13 | 0.3 | Coix seed |  |
| 29 | 2-Monoolein | 34.23 | 0.29 | Coix seed |  |
| 30 | Mandenol | 42.00 | 0.19 | Coix seed |  |


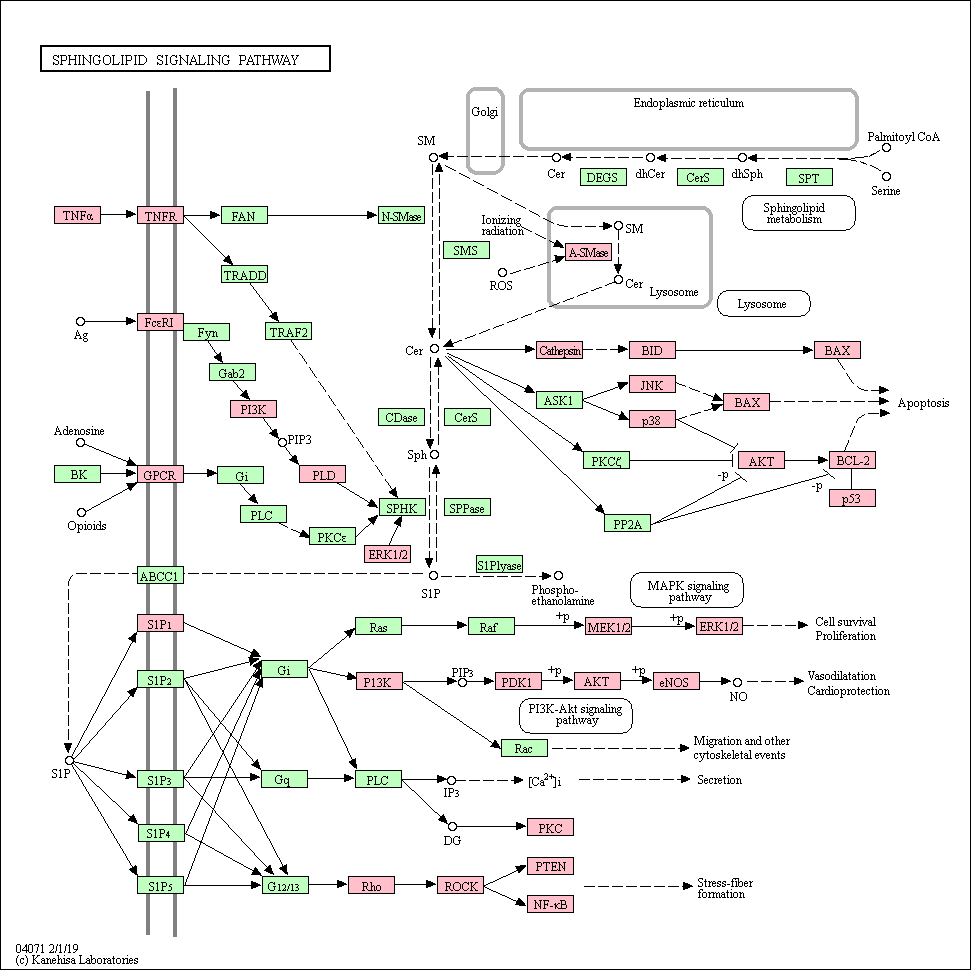


**Figure S1.**Visualization of KEGG pathway of sphingolipid signaling pathway enriched by comorbid proteins of two diseases
